# Cellular and molecular maturation in fetal and adult ovine calcaneal tendons

**DOI:** 10.1111/joa.12269

**Published:** 2014-12-25

**Authors:** Valentina Russo, Annunziata Mauro, Alessandra Martelli, Oriana Di Giacinto, Lisa Di Marcantonio, Delia Nardinocchi, Paolo Berardinelli, Barbara Barboni

**Affiliations:** Faculty of Veterinary MedicineTeramo, Italy

**Keywords:** blood vessels, calcaneal tendon, connexins, extracellular matrix molecules, fetus, growth factors, nerve fibers, pluripotency stem cell markers

## Abstract

Processes of development during fetal life profoundly transform tendons from a plastic tissue into a highly differentiated structure, characterised by a very low ability to regenerate after injury in adulthood. Sheep tendon is frequently used as a translational model to investigate cell-based regenerative approaches. However, in contrast to other species, analytical and comparative baseline studies on the normal developmental maturation of sheep tendons from fetal through to adult life are not currently available. Thus, a detailed morphological and biochemical study was designed to characterise tissue maturation during mid- (2 months of pregnancy: 14 cm of length) and late fetal (4 months: 40 cm of length) life, through to adulthood. The results confirm that ovine tendon morphology undergoes profound transformations during this period. Endotenon was more developed in fetal tendons than in adult tissues, and its cell phenotype changed through tendon maturation. Indeed, groups of large rounded cells laying on smaller and more compacted ones expressing osteocalcin, vascular endothelial growth factor (VEGF) and nerve growth factor (NGF) were identified exclusively in fetal mid-stage tissues, and not in late fetal or adult tendons. VEGF, NGF as well as blood vessels and nerve fibers showed decreased expression during tendon development. Moreover, the endotenon of mid- and late fetuses contained identifiable cells that expressed several pluripotent stem cell markers [Telomerase Reverse Transcriptase (TERT), SRY Determining Region Y Box-2 (SOX2), Nanog Homeobox (NANOG) and Octamer Binding Transcription Factor-4A (OCT-4A)]. These cells were not identifiable in adult specimens. Ovine tendon development was also accompanied by morphological modifications to cell nuclei, and a progressive decrease in cellularity, proliferation index and expression of connexins 43 and 32. Tendon maturation was similarly characterised by modulation of several other gene expression profiles, including *Collagen type I*, *Collagen type III*, *Scleraxis B*, *Tenomodulin*, *Trombospondin 4* and *Osteocalcin*. These gene profiles underwent a dramatic reduction in adult tissues. Transforming growth factor-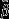
1 expression (involved in collagen synthesis) underwent a similar decrease. In conclusion, these morphological studies carried out on sheep tendons at different stages of development and aging offer normal structural and molecular baseline data to allow accurate evaluation of data from subsequent interventional studies investigating tendon healing and regeneration in ovine experimental models.

## Introduction

Adult tendons are highly differentiated structures; they do not heal completely as they do not activate any regenerative process if injured (Sharma & Maffulli, 2005, 2006; Wang, 2006). Indeed, injured adult tendons can be repaired exclusively through scar formation (Woo et al. 2000; Fenwick et al. 2002; Lin et al. 2004); and, in comparison with normal tendon, they are biomechanically inferior (Lin et al. 2004; Manske, 2005; Tang, 2005). This reduced regenerative property is peculiar to adult tendons as fetal ones are able to regenerate in a strictly age-related manner (Baker & Blair, 1968; Stalling & Nicoll, 2008). Indeed, fetal tissues in the early and mid-gestational stage respond to injuries more efficiently (Holm-Pedersen & Viidik, 1972; Stalling & Nicoll, 2008). Several studies have demonstrated that fetal and adult tendon differences in regenerative properties depend on a complex interaction between extrinsic and intrinsic tenocyte properties. Extrinsic factors influencing tendon healing may involve changes in the precursor cell niche, or differences in local and/or systemic signals. However, the role of cell intrinsic pathways cannot be excluded (Favata et al. 2006; Silva & Conboy, 2008; Piccin & Morshead, 2010).

Barboni et al. (2012a) recently demonstrated that adult and fetal tendons are characterised by different secretory activities. They proved that amniotic epithelial stem cells (AECs) can be differentiated *in vitro* toward tendon cell lineage by co-culturing them with tenocytes/tendon explants that release inductive tenogenic soluble factors. However, these paracrine molecules are significantly more effective if obtained from fetal samples. Even though the mechanisms involved are still unknown, these data strongly indicate that fetal tendon plasticity may depend on a local secretion of paracrine factors that explains the conservation of fetal tendon regenerative properties. Many growth factors have been involved in fetal regeneration, such as transforming growth factor-β (TGF-β), which plays a crucial role in many different cell functions, such as apoptosis, differentiation, epithelial–mesenchymal cell transition, proliferation and extracellular matrix (ECM) production (Schiller et al. 2004; Chen et al. 2005). However, as development progresses from early gestation to the postnatal period, different levels of key growth factors and cytokines may be important in promoting the scarless phenotype (Chen et al. 2005). In addition to the several paracrine activities shown by fetal tendons, fetal tenocytes also have an enhanced cellular migration and collagen production that may support their higher healing capabilities (Stalling & Nicoll, 2008). These intrinsic fetal regenerative properties are shown by data on matrix composition and on tenocytes synthetic activity modifications correlated to tendon maturation and aging (Ippolito et al. 1980; Birch et al. 1985; Moore & De Beaux, 1987; Merkel et al. 1988; DePalma et al. 1989; Whitby & Ferguson, 1991; Batson et al. 2003; Goodman et al. 2004; Stanley et al. 2007; Dunkman et al. 2013). Indeed, a high level of cellularity and of connexins (Cxs) expression was described in immature tendons. Cx43 and Cx32 are involved in tendon gap junction communication, and are highly conserved molecules demonstrated by equine, avian and rat tendons (McNeilly et al. 1996; Ralphs et al. 1998; Waggett et al. 2006; Stanley et al. 2007; Young et al. 2009). Moreover, collagen fibers increase in diameter and vary in thickness with aging. These morphological changes correspond to biochemical modifications that induce an increase in collagen synthesis, a decrease in mucopolysaccharides and in water content (Ippolito et al. 1975, 1980; Shadwick, 1990; Kannus & Józsa, 1991; Birk et al. 1997a,b; Tuite et al. 1997; Hulmes, 2002; Zhang et al. 2005; Kadler et al. 2008; Bruckner, 2010). However, most of the studies available on tendon organisation and function refer to mice, rats, rabbits and horses (Ippolito et al. 1980; Birch et al. 1985; Moore & De Beaux, 1987; Merkel et al. 1988; DePalma et al. 1989; Whitby & Ferguson, 1991; Batson et al. 2003; Goodman et al. 2004; Stanley et al. 2007; Dunkman et al. 2013), while studies regarding ovines are still incomplete. Actually, sheep are frequently used for musculoskeletal regenerative experiments because of their high translational value due to their similarities with humans in terms of weight and mechanical exertion (Wagner & Storb, 1996; Bruns et al. 2000; McCarty et al. 2009). Thus, an extended study on sheep tendon is needed in order to understand tendon morphological and biochemical maturation from fetal through to adult life. Moreover, this research provides morphofunctional elements to accurately evaluate sheep tendon healing and the effectiveness of tested therapeutic approaches.

Based on these assumptions, the present study was designed to analytically describe the morphological and molecular organisation of the ovine tendon in mid (2 months of pregnancy: 14 cm of length) and late (4 months of pregnancy: 40 cm of length) gestation fetuses compared with adult tissues. In particular, it studied the remodeling of cells, ECM and its supporting tissues (blood vessels and nerve endings). Finally, because fetal tendons show intrinsic regenerative properties, their stem modification during tendon development was investigated by analyzing pluripotency stem cell markers, such as Telomerase Reverse Transcriptase (TERT), Nanog Homeobox (NANOG), Octamer Binding Transcription Factor-4A (OCT-4A), SRY Determining Region Y Box-2 (SOX2), and were then compared with adult tissues. This comparative study of fetal tendons vs. adult ones may offer new baseline data on sheep tendon modifications that occur during maturation and aging, as previously demonstrated for other species. These baseline data offer normal structural and molecular information to accurately evaluate sheep tendon healing.

## Materials and methods

### Specimens

Adult and fetal calcaneal tendons were obtained from slaughtered sheep. Adult tendons were collected from 2- to 3-year-old female sheep of the Appenninica breed (*n *= 5). Fetal tendons were isolated from fetuses of mid- (approximately 2 months of pregnancy, 14 cm of length, *n *= 17) and late (approximately 4 months of pregnancy, 40 cm of length, *n *= 21) gestational age (Barone, 1994). Calcaneal tendons were isolated from the hind limbs and deprived of paratenon using a stereomicroscope. Small pieces (about 1 cm^3^ in size) of fresh tissues isolated from the middle portion of calcaneal tendons were immediately cryopreserved for histological hematoxylin-eosin (HE), immunohistochemical (IHC), Western blot and reverse transcriptase-polymerase chain reaction (RT-PCR) analysis.

### HE stain

Samples were cut in serial longitudinal cryosections, 7 μm thick, that were transferred to glass slides. Sections were rehydrated with water and the nuclei stained with an alum hematoxylin (Sigma, St Louis, MO, USA) for 5 min. Then, slides were immersed in tap water for 5 min. The cytoplasm was counterstained with eosin (Sigma, St Louis, MO, USA) for 5 min, and then rinsed well with distilled water. Finally, the sections were dehydrated with ethanol, cleared with xylene and mounted with a resinous medium.

### IHC

Immunohistochemical analyses were carried out on all collected specimens performed with the previous validated antibodies for sheep (Table1). Immunostaining was performed on tissue slides fixed for 10 min with 4% paraformaldehyde/phosphate-buffered saline (PBS). After washing with PBS, non-specific binding was blocked incubating the sections at room temperature (RT) in PBS/1% bovine serum albumin (BSA) for 1 h. Tissue sections were incubated with the primary antibody (Ab) overnight (O/N) at RT, and then exposed to the secondary Ab for 40 min at RT.

**Table 1:** Details of primary and secondary antibodies used for IHC

Primary Abs (Company details)	Primary Ab dilutions	Secondary Abs (Company details)	Secondary Ab dilutions
Ki-67 (Dako Cytomation, Denmark)	1 : 50	Alexa Fluor 488 anti-mouse (Invitrogen, Paisley, UK)	1 : 200
Barboni et al. (2012a,b)			
Cx43 (Chemicon Int. Billrerica, MA, USA)	1 : 200	Alexa Fluor 488 anti-mouse (Invitrogen, Paisley, UK)	1 : 500
Barboni et al. (2012a)			
Cx32 (Chemicon Int. Billrerica, MA, USA)	1 : 200	Alexa Fluor 488 anti-mouse (Invitrogen, Paisley, UK)	1 : 750
Barboni et al. (2012a)			
COL1 (Chemicon Int. Billrerica, MA, USA)	1 : 100	Alexa Fluor 488 anti-mouse (Invitrogen, Paisley, UK)	1 : 200
Barboni et al. (2012a,b)			
COL3 (Chemicon Int. Billrerica, MA, USA)	1 : 500	Alexa Fluor 488 anti-mouse (Invitrogen, Paisley, UK)	1 : 200
Barboni et al. (2012b)			
TGF-β1 (Abcam, Cambridge, UK)	1 : 100	Alexa Fluor 488 anti-mouse (Invitrogen, Paisley, UK)	1 : 200
Barboni et al. (2012b)			
VWF (Dako Cytomation, Denmark)	1 : 400	Alexa Fluor 488 anti-rabbit (Invitrogen, Paisley, UK)	1 : 200
Barboni et al. (2012b, 2013)			
VEGF (Novus Biologicals, Littleton, CO, USA)	1 : 10	Alexa Fluor 488 anti-rabbit (Invitrogen, Paisley, UK)	1 : 200
Barboni et al. (2012b, 2013)			
NF200 (Sigma-Aldrich, St. Louis, MO, USA)	1 : 500	Alexa Fluor 488 anti-rabbit (Invitrogen, Paisley, UK)	1 : 250
Barboni et al. (2014)			
NGF (Sigma-Aldrich, St. Louis, MO, USA)	1 : 400	Alexa Fluor 488 anti-rabbit (Invitrogen, Paisley, UK)	1 : 250
Barboni et al. (2002), Mattioli et al. (1999)			
OCN (Abcam, Cambridge, UK)	1 : 50	Alexa Fluor 488 anti-mouse (Invitrogen, Paisley, UK)	1 : 400
Barboni et al. (2012a, 2013)			
TERT (Calbiochem, Gibbstown, NJ, USA)	1 : 250	Alexa Fluor 568 anti-rabbit (Invitrogen, Paisley, UK)	1 : 250
Russo et al. (2007), Mauro et al. (2010), Barboni et al. (2012a)			
SOX2 (Abcam, Cambridge, UK)	1 : 200	Alexa Fluor 488 anti-rabbit (Invitrogen, Paisley, UK)	1 : 200
Barboni et al. (2012a)			
NANOG (Millipore, Billerica, MA, USA)	1 : 500	Alexa Fluor 488 anti-rabbit (Invitrogen, Paisley, UK)	1 : 200
Barboni et al. (2012a, 2014)			

Primary and secondary antibodies (Abs) were diluted in PBS supplemented with 1% BSA. Cx, connexin; NGF, nerve growth factor; NF200, neurofilament 200; TGF, transforming growth factor; VEGF, vascular endothelial growth factor.

Double-labeling to identify endothelial cells, marking the Von Willebrand factor, and vascular endothelial growth factor (VEGF) was performed according to Martelli et al. (2009). In brief, after the inhibition of endogenous peroxidases, non-specific binding was blocked incubating the sections at RT in normal goat serum (NGS). Sections were then incubated with the rabbit anti-VEGF diluted 1 : 10 in PBS/1% BSA at RT-O/N. After washing, this immunocomplex layer was detected by an anti-rabbit Cy3 (Sigma-Aldrich, Missouri, USA) diluted 1 : 200 in PBS. Furthermore, after washing in PBS/NGS, the same sections were incubated with a rabbit anti-vWF at RT-O/N. After washing in PBS, tissue sections were then processed with an Alexa Fluor 488-labeled secondary goat anti-rabbit Ab. At the end of each immunoreaction, DNA was counterstained with DAPI (Sigma) diluted 1 : 100 in PBS for 10 min. In all experiments, non-immune serum was used in place of the primary antisera as a negative control. All controls performed were negative.

### Morphometric analyses

Morphometric analyses were carried out with an Axioscop 2plus epifluorescence microscope (Zeiss, Oberkochen, Germany) equipped with a cooled color charge-coupled device camera (CCD; Axiovision Cam, Zeiss) interfaced with an interactive and automatic image analyser (Axiovision, Zeiss). Serial tissue sections were analyzed under 200 × magnification. The quantification of digitised signals was completed using a semi-automated algorithm of the image analysis software KS300 (Zeiss), as previously described (Martelli et al. 2009; Barboni et al. 2012b). Guided programs (macros for KS300) were created to count internally a standard field of 15 000 μm^2^:

1. cellularity (total number of DAPI-stained nuclei in the field; Martelli et al. 2009; Barboni et al. 2012b);
2. proliferation index (PI; % Ki-67 green-stained cells/total number of nuclei in the field; Martelli et al. 2009; Barboni et al. 2012b), quantified to evaluate cell proliferation;
3. TGF-β1 expression (TGFβ, positive area in μm^2^ in the field; Marks et al. 2006), to measure the ECM remodeling key factor;
4. neurofilament 200 (NF200) expression (NF200, positive area in μm^2^ in the field; Ferreira-Gomes et al. 2010; Chen et al. 2013) to evaluate tendon innervation;
5. nerve growth factor (NGF) expression (NGF, positive area in μm^2^ in the field; Fressinaud et al. 2003), to measure the key molecule for nerve growth and maintenance;
6. vascular area (VA, vWF-positive area in μm^2^ in the field; Martelli et al. 2009; Barboni et al. 2012b), to evaluate blood vessel organisation;
7. VEGF expression (VEGF, positive area in μm^2^ in the field; Maae et al. 2011), to measure the key molecule for blood vessel remodeling.

In particular, the quantification of the digitised fluorescent signals was accomplished after the background densitometry calibration, the algorithm detected and separated the fluorescent regions of interest from their background and created a new image on the basis of their gray values. Thus, the process created a binary image from true color images. The analyses were performed on the whole area of the cross-section (Martelli et al. 2009; Barboni et al. 2012b).

The analyses were carried out on at least three serial sections/calcaneal tendons and at least two different fields/sections for each analysis.

### Western blot analysis

Proteins were extracted from small pieces of fetal and adult calcaneal tendons (about 1 cm^3^ in size) and processed according to Mauro et al. (2014). Proteins (75 μg) were then separated by 10% sodium dodecyl sulfate–polyacrylamide gel electrophoresis and electrophoretically transferred to a nitrocellulose membrane (Hybon C Extra; Amersham Pharmacia, Piscataway, NJ, USA) for Western blot analysis (Towbin et al. 1979). Protein detection was performed by incubating membranes with the monoclonal anti-Cx32 and anti-Cx43 primary antibodies (1 : 200 dilution), and monoclonal anti α-tubulin (1 : 500; Sigma, St Louis, USA). Goat anti-mouse immunoglobulins peroxidase-conjugated (IgG-HRP; 1 : 1000; Santa Cruz Biotechnology, Heidelberg, Germany) were finally used as secondary antibodies. The signals were detected using the ECL Western Blot Analysis System (Amersham Pharmacia, Amersham Pharmacia, Piscataway, NJ, USA). Cx32 and Cx43 protein expression quantitative data were determined as the mean ratio of the optical density of the specific bands normalised for the α-tubulin expression and carried out with the Advanced Image Data Analyser (Rai Test; GMBH, Straubenhardt, Germany).

### Total RNA isolation and RT-PCR

Reverse transcriptase-PCR analyses were performed as previously described (Barboni et al. 2012a,b) on ovine mid- and late-gestation fetuses, and adult tendons, to compare the expression of the genes summarised in Table2.

**Table 2:** Primer sequences used for RT-PCR

Gene	Accession number	Primer sequence	Product size (bp)	PCR cycles
*GADPH*	AF030943.1	F: CCTGCACCACCAACTGCTTG	224	40
	Ovine	R: TTGAGCTCAGGGATGACCTTG		
*COL1*	AF129287.1	F: CGTGATCTGCGACGAACTTAA	212	40
	Ovine	R: GTCCAGGAAGTCCAGGTTGT		
*COL3*	AY091605.1	F: AAGGGCAGGGAACAACTTGAT	355	40
	Ovine	R: GTGGGCAAACTGCACAACATT		
*TNMD*	NM_001099948.1	F: TGGTGAAGACCTTCACTTTCC	352	40
	Bos Taurus	R: TTAAACCCTCCCCAGCATGC		
*THBS4*	NM_001034728.1	F: CCGCAGGTCTTTGACCTTCT	231	40
	Bos Taurus	R: CAGGTAACGGAGGATGGCTTT		
*SCXB*	XM_866422.2	F: AACAGCGTGAACACGGCTTTC	299	45
	Bos Taurus	R: TTTCTCTGGTTGCTGAGGCAG		
*OCN*	DQ418490.1	F: AGACACCATGAGAACCCCCAT	234	40
	Ovine	R: TTGAGCTCACACACCTCCCT		
*NANOG*	FJ970651.1	F: TGGATCTGCTTATTCAGGACAG	209	40
	Ovine	R: TGCTGGAGGCTGAGGTATTTC		
*SOX2*	X96997.1	F: ACCAGAAGAACAGCCCGGAC	264	45
	Ovine	R: TCATGAGCGTCTTGGTTTTCCG		
*TERT*	EU139125.1	F: TTGTCCCCGCAGGTGTCTTG	176	45
	Ovine	R: TGACCGTGTTGGGCAGGTAG		
*OCT4A*	NM_174580.1	F: TATGACTTGTGTGGAGGGATG	327	45
	Bos Taurus	R: AAACAGAACCCCCAGGGTGA		

PCR, polymerase chain reaction.

Total RNA was extracted using TRI Reagent (Sigma-Aldrich, Missouri, USA) according to the manufacturer's instructions. RNA integrity and size distribution were evaluated by 1% agarose gel electrophoresis and ethidium bromide staining. Digestion of genomic DNA was carried out by DNaseI digestion (Sigma-Aldrich, Missouri, USA) for 15 min at RT. One microgram of total RNA of each sample was used for RT reaction with Oligo dT primer and BioScriptTM Kit (Bioline, London, UK); 2 × ReadyMix^TM^ Taq PCR reaction mix (Sigma-Aldrich, Missouri, USA) was used for PCR reaction using 3 μL of cDNA and 0.5 μm of each primer, in a final volume of 25 μL. The primer sequences, Genebank number of reference mRNA sequences, product length and cycles are shown in Table2. The PCR reaction mixtures were incubated for 5 min at 95 °C, followed by 95 °C for 30 s, 55 °C for 30 s, 72 °C for 45 s and 72 °C for 7 min. For each gene, a reaction mixture with water instead of cDNA template was run at the same time, as a PCR-negative control. RT-PCR was normalised by the transcriptional levels of GAPDH. The PCR products were separated on 2% agarose gel stained with ethidium bromide, visualised on a Gel Doc 2000 (Bio-Rad, Hercules, CA, USA) and analyzed with Quantity One 1-D Analysis software (Bio-Rad, Hercules, CA, USA). Each PCR reaction was carried out in triplicate.

As a positive control (CTR-positive) for the pluripotency stemness gene expression profile, freshly isolated ovine (o)AECs were used (Barboni et al. 2012a,b). In brief, oAECs were collected from slaughtered sheep pertaining to Appenninica breed at 3 months of pregnancy (25–30 cm in length) according to Barboni et al. (2012a). Once the uterus wall was opened, the oAECs were collected from pieces of approximately 3–5 cm of the amniotic membranes that were mechanically peeled off. Membrane pieces were washed in PBS (Sigma Chemical, St Louis, MO, USA), and incubated in 0.25% Trypsin/EDTA 200 mg L^−1^ at 37.5 °C for 20 min under gentle agitation. Then, cell suspension was collected, filtered through a 40-mm cell filter and poured into a 50-mL falcon tube containing fetal calf serum at a final concentration of 10% to inactivate trypsin. Each falcon tube was centrifuged, and the pelleted cells were then used for the RT-PCR analyses.

### Statistical analysis

The quantitative data obtained, expressed as mean ± standard error (± SE), were first assessed for normalcy of distribution by D'Agostino and Pearson test. Data sets were compared using anova test followed, when necessary, using the *post hoc* Tukey test (GraphPad Prism 5, GraphPad Software, USA). The values were considered statistically significant for *P *≤ 0.05.

## Results

### Fetus and adult tendon histology

**Figure 1:**
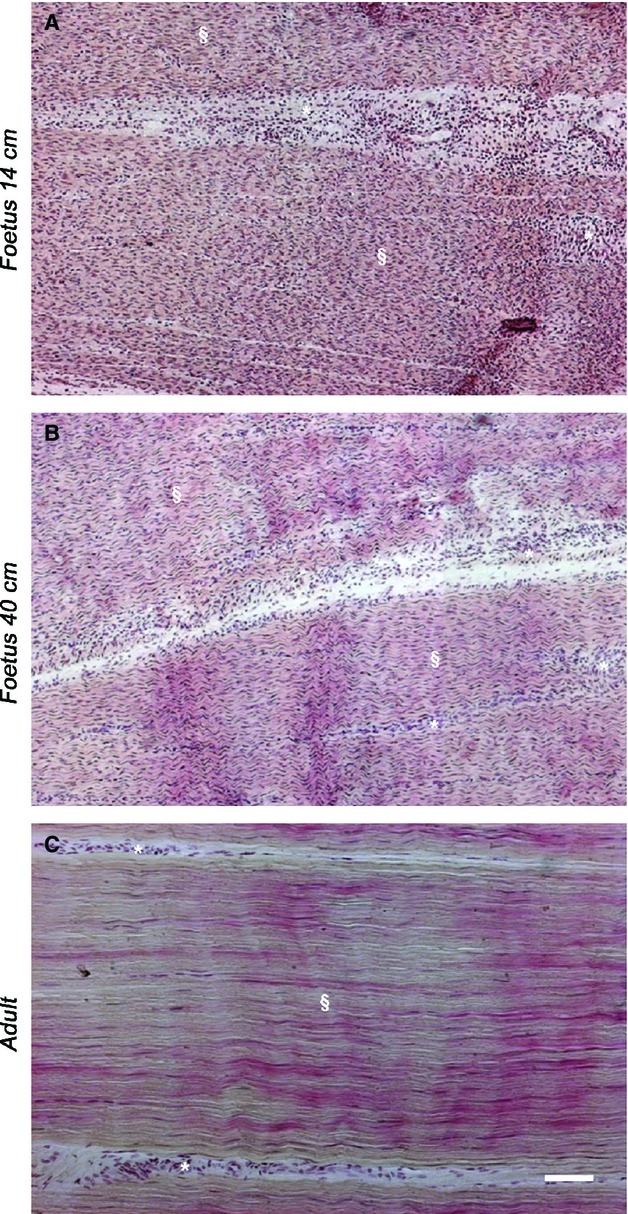
Representative micrographs of HE staining in analyzed tendons. (A) Fourteen-centimeter fetus (mid-pregnancy), (B) 40-cm fetus (late pregnancy) and (C) adult tendon. In fetal tendons (A, B), the endotenon (*) was more developed and thicker (especially in the mid stage fetus one), (A) than that observed in adult tissue (C). Tendon tissue proper (§) is indicated in fetal (A, B) and adult tendons (C). Scale bar: 50 μm.

General morphology of all the analyzed tendons (mid-gestation: 14 cm; late gestation: 40 cm fetuses; and adult tissues) was shown with HE staining (Fig.1). Calcaneal tendon specimens, deprived of their paratendinous connective tissue, consisted of tendon tissue proper and endotenon connective tissue. The borderline between the two tissue types was readily defined microscopically. Tendon tissue proper modified its organisation during tendon development, acquiring in adult tissues the final morphology that consisted of large groups of collagen fibrils lying in parallel with the tenocytes visible between them. No evidence of nuclear picnosis or nuclear fragmentation was observed in any analyzed samples. Endotenon was recorded within the tendon: it was composed of loose connective tissue containing dispersed rounded cells. The endotenon was more developed and thicker in both types of fetal tendons, especially in mid-stage ones, than in adult tissues (Fig.1A,C).

### Tenocyte nuclei morphology, cellularity and PI in fetuses and adults

**Figure 2:**
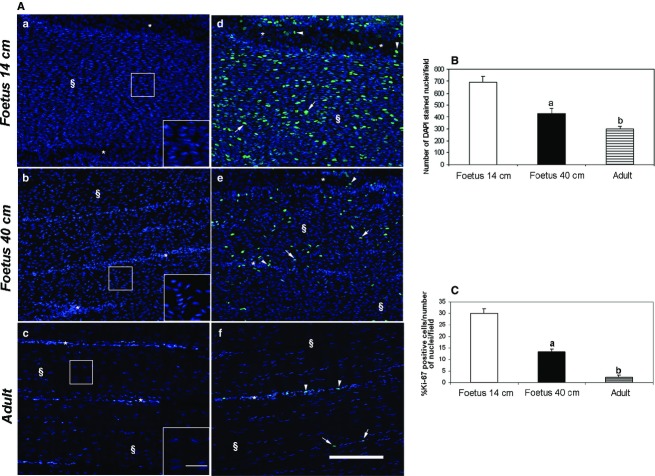
Representative micrographs of tenocyte nuclei morphology, cellularity and PI quantification in fetus and adult tendons. Thicker endotenon (*) and tendon tissue proper (§) are indicated in the images. (A) On the left panel are shown: cell nuclei (DAPI blue stain) morphology derived from fetuses of mid (a), late pregnancy (b) and adult tissues (c). In the box, details of tenocyte nuclei shapes in tendon tissue proper (§), scale bar: 25 μm. Ki-67, a cell proliferation marker, positive cells (green stained), in fetuses of mid (d), late pregnancy (e) and adult tissues (f). Cell nuclei were counterstained with DAPI (blue stain). Arrowheads and arrows indicate examples of Ki-67-positive cells in endotenon (*) and tendon tissue proper (§), respectively. Scale bar: 50 μm. (B) The histograms indicate cellularity in fetuses of mid, late pregnancy and adult tendons determined as the mean ± standard error (± SE) of the total number of DAPI-stained nuclei in the field. ^a^Significantly different values between fetuses (*P *< 0.05); ^b^significantly different values of adult samples vs. both types of fetuses (*P *< 0.05). (C) The histograms show the PI of the analyzed tendons as mean ± SE of % Ki-67 green-stained cells/total number of nuclei in the field. ^a^Significantly different values between fetuses (*P *< 0.05); ^b^significantly different values of adult samples vs. both types of fetuses (*P *< 0.05).

Tendons derived from fetuses of 14 cm (mid-gestation) and 40 cm (late pregnancy) showed cell nuclei of variable shapes (elongated, ovoid or polygonal). Tenocyte nuclei assumed a typical spindle shape in adult tissues (Fig. 2Aa–c). They had a round morphology in mid-fetal tendons (Fig. 2Aa), while they assumed an elongated shape and a parallel arrangement along the tissue longitudinal axis in adult tendons (Fig. 2Ac). An intermediate cell morphology was observed in 40-cm fetuses, as cells acquired a more fusiform nuclei appearance and a more evident alignment (Fig. 2Ab).

As shown in Fig.2B, cellularity was higher at the beginning of tendon development (14-cm fetuses) to gradually decrease during fetal (*P *< 0.05, 14 cm vs. 40 cm) and adult life (*P *< 0.05, 40 cm vs. adult). The PI of tendon cells, analyzed by a specific proliferation marker KI-67, was much higher in fetal cells than in adult ones (Fig. 2Ad–Af). In particular, adult tissues showed proliferating cells exclusively in the endotenon, while KI-67-positive cells were observed both in endotenon and tendon tissue proper in fetal tendons (Fig. 2Ad,Ae). Quantitative analysis showed that the percentage of proliferating cells decreased from ∼30% to ∼14% during fetal life (*P *< 0.05), to decrease to 5% in adult tissues (Fig.2C).

### Expression and quantification of Cx43 and Cx32 in fetal and adult tendons

**Figure 3:**
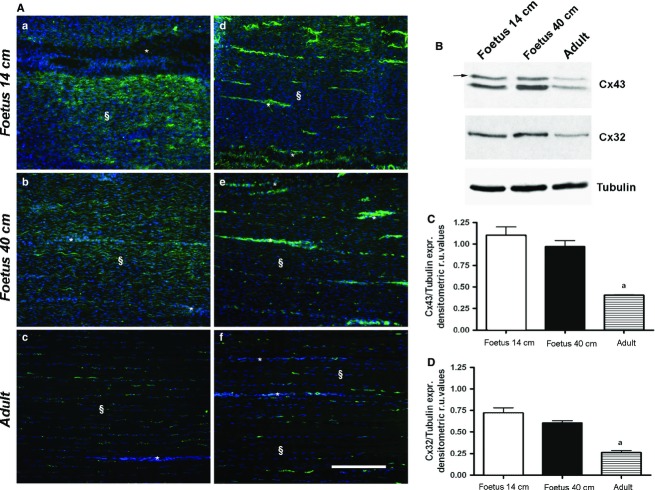
Representative micrograph of Cx43 and Cx32 protein localisation (A), expression (B) and their quantification (C, D) in fetal and adult tendons. In the left panel (A), Cx43 (a–c) and Cx32 (d–f) protein expression (green stain) in the analyzed tendons is shown. The cell nuclei were counterstained with DAPI (blue stain). The tendons were isolated from fetuses of mid (a, d), late (b, e) pregnancy and adult (c, f) tissues. Endotenon (*) and tendon tissue proper (§) are indicated in the images. Scale bar: 50 μm. On the right panel (B), representative images of Cx43 and Cx32 protein expression analyzed by Western blot; the arrow indicates the phosphorylated form of Cx43 protein. Histograms show densitometric values of Cx43 (C) and Cx32 (D) protein expression normalised for α-tubulin expression. The values are expressed as the mean of three independent experiments ± SE for each sample. ^a^Significantly different values of adult samples vs. both types of fetuses (*P *< 0.05).

Both Cx43 and Cx32 proteins were always expressed in the analyzed tendons, even if they showed different expression patterns and localisation (Fig.3). As shown in Fig.3A, both proteins showed a typical punctiform immunoreactivity. Cx43 appeared widespread throughout the tissue (Fig. 3Aa–Ac), while Cx32 involved only neighboring cells placed in a single row (Fig. 3Ad–Af). Both Cx43 and Cx32 proteins showed similar high levels of expression during fetal life (*P *> 0.05) that significantly decreased (*P *< 0.05) in the adult tissue (Fig.3B–D).

### Analysis of the ECM composition

**Figure 4:**
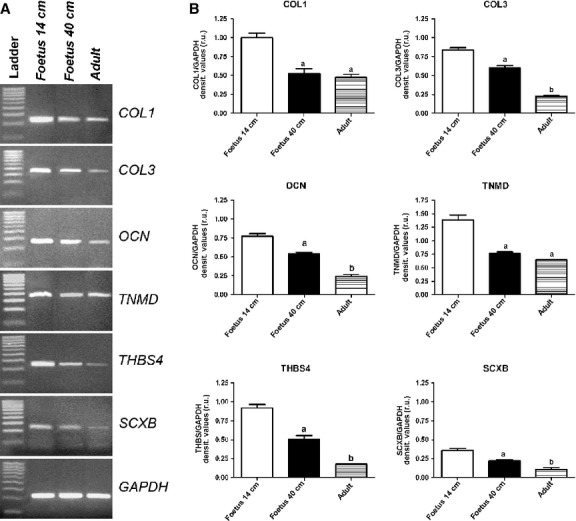
(A) Representative images of *COL1*, *COL3*, *TNMD*, *THBS4*, *SCXB* and *OCN* mRNA gene expression profile by RT-PCR in mid and late fetal, and adult tendons. (B) The histograms indicate a semi-quantitative analysis of gene expression normalised for GAPDH. Each value was expressed as the mean of three replicates ± SE for each sample. ^a^Significantly different values between fetuses (*P *< 0.05); ^b^significantly different values of adult samples vs. both types of fetuses (*P *< 0.05).

The expression of tendon ECM molecules, such as *Collagen type I* (*COL1*), *Collagen type III* (*COL3*), *Scleraxis B* (*SCXB*), *Tenomodulin* (*TNMD*), *Trombospondin 4* (*THBS4*), and *Osteocalcin* (*OCN*) were analyzed during tendon development (Fig.4A). All these genes were overexpressed in mid-fetal tendons (Fig.4B). However, while *COL1* and *TNMD* mRNA expression remained similar during late fetal and adult life*, COL3*, *THBS4*, *SCXB* and *OCN* decreased further, becoming significantly lower in adult tissues (*P *< 0.05; Fig.4B).

**Figure 5:**
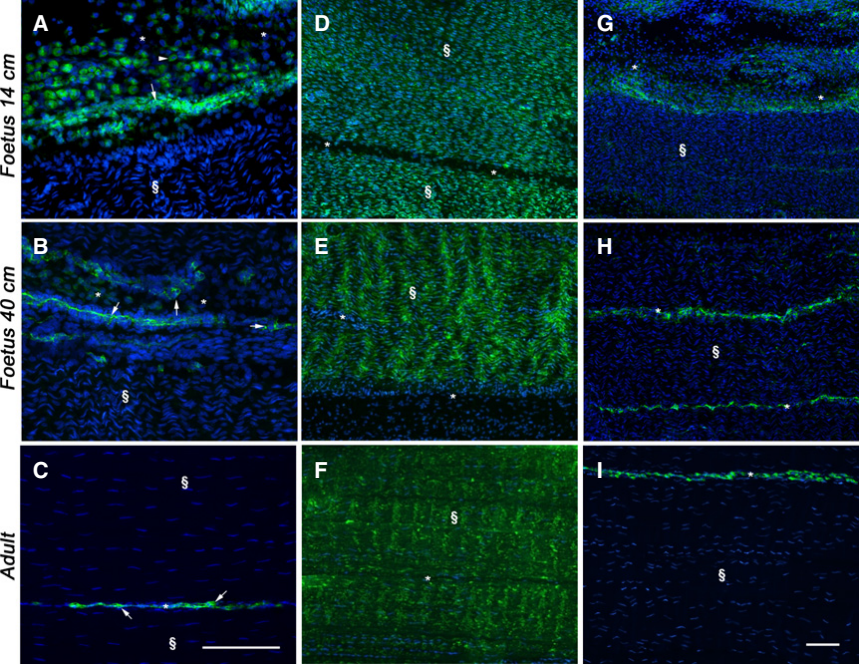
Representative micrographs of OCN, COL1 and COL3 immunostaining in mid (A, D, G) and late (B, E, H) fetal, and adult (C, F, I) tendons. Endotenon (*) and tendon tissue proper (§) are indicated in the images. On the left images (A–C), OCN-positive cells (green stain), with large rounded (arrowhead) or smaller compacted (arrow) morphology, are localised in tendons. Scale bar: 25 μm. The middle (D–F) and the right (G–I) images show COL1 and COL3 proteins (green stain), respectively, in the analyzed tendons. Cell nuclei were counterstained with DAPI (blue stain). Scale bar: 25 μm.

**Figure 6:**
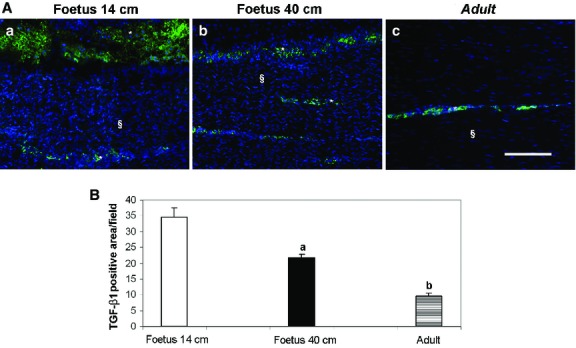
Representative micrographs of TGF-β1 immunostaining (A) and its quantification (B) in the analyzed tendons. Endotenon (*) and tendon tissue proper (§) are indicated in the images. (A) The images of (a) 14-cm fetuses, (b) 40-cm fetuses and (c) adult tendons show how TGF-β1 was always recorded within the endotenon in the ECM during tendon development. Scale bar: 50 μm. (B) The histograms show the quantitative analysis of TGF-β1-positive area in μm^2^ field expressed as mean ± SE. ^a^Significantly different values between fetuses (*P *< 0.05); ^b^significantly different values of adult samples vs. both types of fetuses (*P *< 0.05).

The localisation of *OCN*, *COL1* and *COL3* revealed their profound modifications during tendon development. *OCN* was detected in adult and fetal tendons, and it was expressed only in endotenon cells (Fig.5A–C). In particular, only in the mid-fetal stage of tendon development, two different classes of cells localising *OCN* within their cytoplasm were observed: large rounded cells organised in groups and distributed over a layer of smaller and more compacted cells (Fig.5A). This second type of *OCN*-positive cells persisted in the late fetal and adult tendons. Moreover, in 14-cm fetuses tendons, *COL1* was recorded as an intracytoplasmic protein (Fig.5D), becoming extracellular at the end of fetal life (Fig.5E). At this stage of development, *COL1* proteins appeared at first as oriented fibers and showed a regular parallel orientation in adult tissues (Fig.5F). The endotenon did not show any positivity for *COL1* (Fig.5D–F) whereas, in all analyzed samples, *COL3* was exclusively localised in the endotenon (Fig.5G–I). In particular, in late gestation fetuses, this protein was localised only at the sides of the endotenon (Fig.5H). In order to have additional information on ECM remodeling, the localisation of the key growth factor, TGF-β1, was analyzed. TGF-β1 was always recorded within the endotenon, in particular in the ECM (Fig. 6Aa–c). Quantifying its expression, it was evident that the production of this growth factor was strictly related to the age of the subject (*P *< 0.05; Fig.6B).

### Blood vessel remodeling and VEGF expression

**Figure 7:**
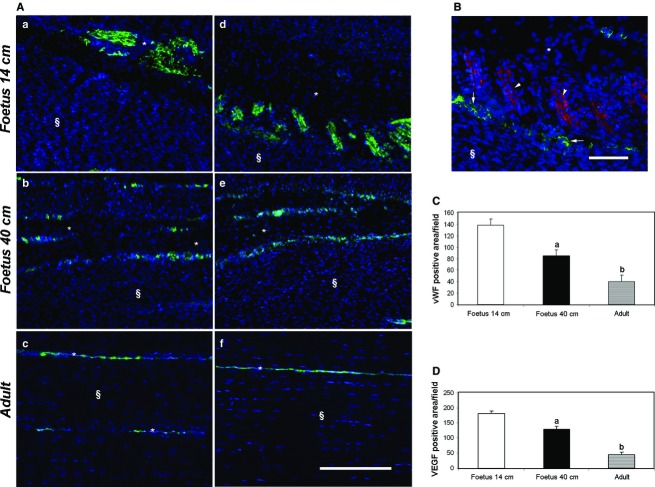
Representative micrographs of vWF and VEGF immunostaining (A, B) in the analyzed tendons and their quantification (C, D). (A) On the left images (a–c), vWF (green stain), an endothelial marker, is shown. On the right images (d–f), VEGF protein (green stain) is localised in the analyzed tendons. Tendons were isolated in fetuses of mid (a, d, B), late (b, e) pregnancy and adults (c, f). Endotenon (*) and tendon tissue proper (§) are indicated in the images. Scale bar: 50 μm. (B) vWF (green stain) and VEGF protein (red stain) distribution within the endotenon visualised using a double-immunostaining. VEGF-secreting cells (arrowed) are closely positioned to vWF endothelial cells (arrow) of blood vessels. Cell nuclei were counterstained with DAPI (blue stain). Scale bar: 25 μm. (C) The histograms show the VA in fetuses of mid- and late pregnancy and adult tendons determined as the mean ± SE of vWF-positive area in μm^2^ field. ^a^Significantly different values between fetuses (*P *< 0.05); ^b^significantly different values of adult samples vs. both types of fetuses (*P *< 0.05). (D) The histograms show VEGF-positive area in μm^2^ field expressed as mean ± SE. ^a^Significantly different values between fetuses (*P *< 0.05); ^b^significantly different values of adult samples vs. both types of fetuses (*P *< 0.05).

Sheep tendon development was actively accompanied by angiogenesis and vascular remodeling. Blood vessels (Fig. 7Aa–c) and expression of the major angiogenic factor, VEGF (Fig. 7Ad–f), were both localised during fetal and adult life within the endotenon. Interestingly, as demonstrated by the dIHC for vWF and VEGF, in 14-cm fetus tendons VEGF-secreting cells were organised in islets that were positioned close to blood vessels (Fig.7B). A close correlation between VEGF and blood vessels was also observed in older tendons (late stage of fetal and adult life), although islets containing VEGF-secreting cells disappeared (Fig. 7Ae,Af). The extension of total VA corresponded to the VEGF expression (Fig.7C,D). Indeed, a linear decrease in VA extension and VEGF expression was recorded with the progression of tendon development: VA and VEGF expression were ∼four times higher and ∼twice as high, respectively, in 14-cm fetus tendons than those recorded in late fetal or adult tendons, respectively (*P *< 0.05).

### Innervation and NGF expression during tendon maturation

**Figure 8:**
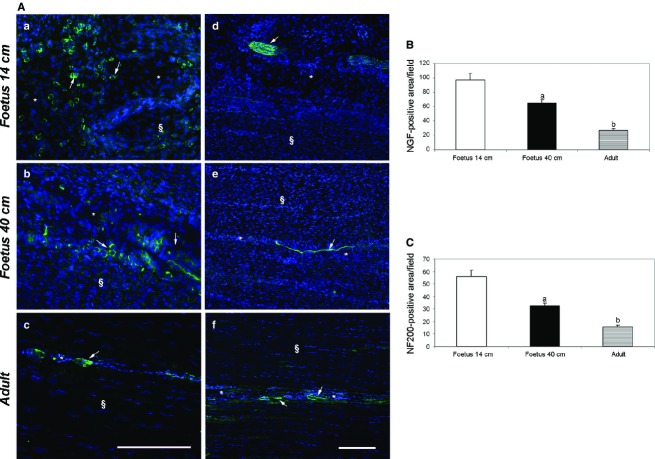
Representative micrographs of NF200 and NGF protein expression (A) and their quantification in fetal and adult tendons (B, C). The tendons were isolated from fetuses of mid- (a, d) and late (b, e) pregnancy and adults (c, f). Endotenon (*) and tendon tissue proper (§) are indicated in the images. (A) On the left images (a–c), NGF protein (green stain) localised in the analyzed tendons is showed. Examples of round NGF-positive cells (arrow) are indicated in endotenon. The right images (d–f) show NF200 protein (green stain) in the analyzed tendons. Example of neurofilaments (arrow) are indicated in the images. Cell nuclei were counterstained with DAPI (blue stain). Scale bar: 50 μm. (B, C) Histograms show quantitative analyses of (B) NGF- or (C) NF200-positive area in μm^2^ field expressed as mean ± SE. ^a^Significantly different values between fetuses (*P *< 0.05); ^b^significantly different values of adult samples vs. both types of fetuses (*P *< 0.05).

A modulation of nerve fibers and NGF expression followed tendon development. In detail, NGF expression was recorded within the cytoplasm of rounded cells widespread in the endotenon of both types of fetuses (Fig. 8Aa,Ab). However, at the mid-stage of fetal tendon development nerve fibers formed large neurofilament conglomerates (Fig. 8Ad). Subsequently, the innervation became lower at the end of gestation (Fig. 8Ae). Adult tendons had NGF positivity in the endotenon having an extracellular localisation (Fig. 8Ac), and a discrete innervation (Fig. 8Af). These morphological data were confirmed by NGF and NF200 quantification, which decreased based on the age of the subject (*P *< 0.05; Fig.8B,C).

### Expression and localisation of stem cell markers in fetal tendons

**Figure 9:**
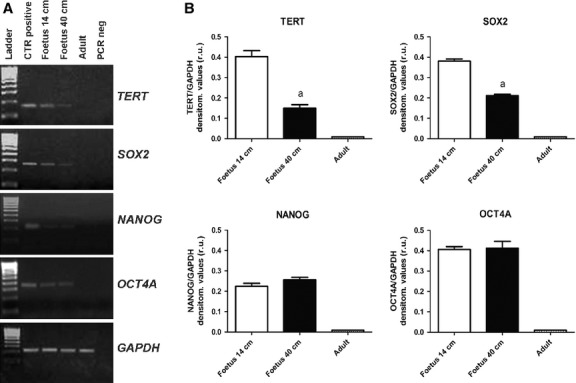
(A) Representative images of *TERT*, *SOX2*, *NANOG* and *OCT-4A* mRNA stemness-related gene expression profile by RT-PCR in 14-cm fetuses, 40-cm fetuses and adult tendons. (B) The histograms indicate a semi-quantitative analysis of gene expression normalised for GAPDH. Each value was expressed as the mean of three replicates ± SE for each sample. ^a^Significantly different values between fetuses (*P *< 0.05); ^b^significantly different values of adult samples vs. both types of fetuses (*P *< 0.05).

*TERT*, *SOX2*, *NANOG* and *OCT-4A* mRNA expression disappeared in adult tendons, while they had a different behavior in fetal tissues (Fig.9A). In particular, *NANOG* and *OCT-4A* expression were stable during fetal life, while *TERT* and *SOX2* progressively decreased (Fig.9B).

Stemness markers immunolocalisation demonstrated that TERT, SOX2 and NANOG proteins were present in fetal tendons, but were totally unexpressed in adult tendons, confirming RT-PCR analysis. Unfortunately, it was not possible to study OCT-4A because sheep-specific Ab is still not commercially available. In particular, TERT, SOX2 and NANOG were recorded within several rounded cells, already described in the endotenon of 14- and 40-cm fetuses. NANOG was always recognised in the nuclei of these positive cells (Fig.10G,H), whereas TERT and SOX2 nuclear positivity involved only a portion of these cells, while some other cells showed a cytoplasmic positivity to these two markers (Fig.10A,B,D,E). No immunoreactivity to these antibodies was recorded in adult tendons (Fig.10C,F,I).

**Figure:**
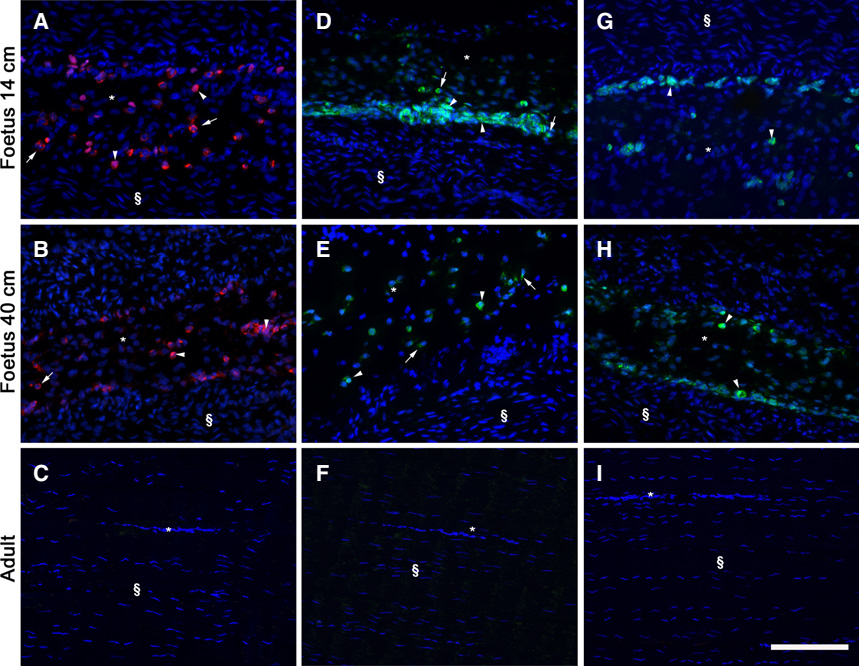
Representative micrographs of immunostaining for TERT, SOX2 and NANOG in mid (A, D and G), late (B, E and H) fetal, and adult tendons (C, F and I). In adult tendons no immunoreactivity to these markers was present. Endotenon (*) and tendon tissue proper (§) are indicated in the images. All these pluripotency stemness markers were always localised within the endotenon (*). On the left images (A, B), TERT localisation (red stain) in the analyzed tendons is shown. The middle images (D, E) show SOX2 distribution (green stain) and, on the right images (G, H), NANOG protein (green stain) expression is also assessed. Examples of nuclei (arrowhead) or cytoplasm (arrow) positive cells are indicated for each marker in the images. Cell nuclei were counterstained with DAPI (blue stain). Scale bar: 50 μm.

## Discussion

Similar to other connective tissues, tendons go through morphological and molecular changes during aging. These modifications involve cells and ECM. Initially a tendon is a high cellularity tissue working in syncytium; in adult life, it becomes a specialised tissue with few cells that have a reduced communication and very low synthetic activity (Ippolito et al. 1980; Young et al. 2009).

Although all these findings are available in animals such as mice, rats, rabbits and horses [Ippolito et al. 1980; Moore & De Beaux, 1987; Merkel et al. 1988; DePalma et al. 1989; Whitby & Ferguson, 1991; Batson et al. 2003; Goodman et al. 2004; Stanley et al. 2007 (horse); Birch et al. 1985; Dunkman et al. 2013], ovine morphological organisation and biochemical matrix composition during tendon maturation has not previously been studied.

The present results demonstrate that sheep tendon maturation is accompanied by a progressive decrease in cell number and by cell morphology specialisation. Indeed, 14-cm fetus tendons had a quite heterogeneous (elongated, rounded or polygonal cells) cell nuclei shape, and their arrangement along the longitudinal axis is not definite. A gradual decrease in tenocyte number occurred in 40-cm fetuses where the cell nuclei started to acquire a spindle-shaped morphology. Cellularity further decreased in adults to eventually acquire the final elongated shape of the nuclei. Thus, the number of tenocyte/field resulted significantly greater in immature sheep than in adult ones, as demonstrated in horses (Stanley et al. 2007). In sheep tendons it can be hypothesised that during aging, cell reduction is not due to apoptosis; in fact, no evidence of nuclear picnosis or nuclear fragmentation was observed. Similarly, a low incidence of apoptotic cells was demonstrated in normal adult horse superficial digital flexor tendon (SDFT; Hosaka et al. 2004) and in adult human patellar tendons (Chuen et al. 2004). On the basis of this evidence, the higher cellularity shown in fetal tendons may be attributable to a greater PI of fetal tenocytes and to a smaller accumulation of the ECM. These findings are coherent with the conventional view that adult tenocytes are differentiated cells with a greater cell/ECM ratio and a very limited proliferative capacity, as also demonstrated by Chuen et al. (2004) in humans. Probably the low PI in adult tendons is not only a consequence of the complete tissue growth but is also due to an intrinsic functional limiting condition of the adult tissue. Indeed, it has been demonstrated that sheep adult tendons were not able to modulate their PI if stimulated or stressed. In fact, after an experimental injury, sheep adult tendons slightly increased their proliferative capacity, whereas it was significantly improved after stem cell transplantation (i.e. oAECs; Barboni et al. 2012b). Previous studies carried out in fetal tissues (Becker & Mobbs, 1999; Becker et al. 1999) have shown that PI is positively regulated by gap junction expression. Cx43 and Cx32 expression in sheep, as in horses (Stanley et al. 2007), were significantly higher in fetal tendons. On the other hand, as suggested by other authors (Koterba, 1990; Lin et al. 2005), this greater cellular population may be required until the late stage of gestation to facilitate the rapid adaptive organisation of matrix components during the first few days of life, as well as to support a correct ECM deposition during postnatal life. Gap junctions have a conserved organisation amongst species. Indeed, in this research Cx32 was localised between tenocytes of the same row, whereas Cx43 connected cells were located amongst different rows, as previously described in tendons of other animal models (McNeilly et al. 1996; Ralphs et al. 1998; Stanley et al. 2007). Gap junction communication is essential to create networks amongst tenocytes that can exchange ions and small molecules (< 1 kDa) guaranteeing the electrical coupling (Willecke et al. 2002), and facilitating the diffusion of signaling and nutrients in this poorly vascularised tissue (Tanji et al. 1995). Gap junction communication in tendons allows coordination of synthetic responses to mechanical stimuli (i.e. mechanotransduction). Indeed, evidence shows that Cx43 may be important in tendon cell response to mechanical load: cultured tendon cells exposed to tensile loading upregulate Cx43 expression, and use these gap junction protein channels to propagate calcium fluxes from cell to cell when mechanically stimulated (Banes et al. 1995). This function was also associated with strain-induced collagen synthesis (Amiel et al. 1995; Banes et al. 1999; Ko & McCulloch, 2001). Indeed, chemical gap junction blockers in fibroblasts, including tenocytes, produced a significant reduction in type I and total collagen synthesis (Banes et al. 1999; Ehrlich et al. 2006). Given the role of gap junctions in the facilitation of tenocyte collagen synthesis, a decline in Cx expression measured in the adult tendon supports the hypothesis that tenocyte networks in adults are less responsive in regenerating mechanical strain and/or microdamage. Even though levels of *COL1* mRNA in sheep remained similar during late fetal and adult life, its expression was greatly decreased compared with mid-stage fetuses. However, in mid-stage fetuses, *COL1* upregulation corresponded to a cytoplasmic distribution of the protein. It can be hypothesised that at this stage this molecule is not an active component of the ECM. Thus, the decrease in gap junction expression and collagen synthesis that occurs during sheep tendon maturation and growth supports the hypothesis that tenocyte population in adult tendons is less active in molecular synthesis, as previously demonstrated in the equine SDFT (Young et al. 2009). A greater COL1 and COL3 synthetic activity of immature tenocytes was also observed in equine SDFT in response to cyclical strain and TGF-β stimulation (Goodman et al. 2004). Similarly, TGF-β in sheep appeared to be related to COL1 synthesis and remodeling that accompanied tendon maturation: it was, indeed, more present in tissues of mid-gestation fetuses than in the late ones, eventually becoming permanent at low levels in adult calcaneal tendons. The modification of the expression profile also involved the other ECM genes such as *SCXB*, *TNMD*, *THBS4*: they were always overexpressed in fetal respect to adult tendons. Amongst the genes being considered, the higher expression of *SCXB* promoted in mid-fetal samples appeared to be the most remarkable event, considering that *SCXB* is the best characterised tendon neoformation molecular marker (Schweitzer et al. 2001; Murchison et al. 2007). *SCXB*, in fact, is a member of the basis-helix-loop-helix super-family of transcription factors involved in development processes such as tendon formation and tendon muscle attachment during fetal life (Cserjesi et al. 1995). *SCXB* basal expression in adult tendons suggests its positive role in homeostasis as co-activator of other tendon-related genes, such as *COL1* (Léjard et al. 2007) and *TNMD* (Shukunami et al. 2006). Indeed, *TNMD* is necessary to ensure the proper collagen network formation and tendon organisation (Liu et al. 2011), and *COL1* mRNA expression remained similar during late fetal and adult life. *OCN* expression decreased during tendon maturation, becoming significantly lower in adult tissues. Although *OCN* is considered an osteogenic marker, its expression was previously demonstrated in sheep fetal and adult tendons, and in tendon-like structures obtained after oAECs differentiation towards tenogenic lineage (Barboni et al. 2012a,b). This research confirmed the *OCN* mRNA expression in the mid-portion of the analyzed calcaneal tendons, and the presence of the protein in some cells localised in the endotenon, especially in the mid-gestation fetuses. Despite the *OCN* expression in the analyzed tendons, it was not observed in tissue ossification. It was recently demonstrated that *SCXB* increases teno-lineage differentiation but inhibits osteo-lineage differentiation (Alberton et al. 2012; Chen et al. 2012) by antagonising BMP signaling cascade (Chen et al. 2012). In addition, Hoffmann et al. (2006) demonstrated that C3H10T1/2-BMP2/Smad8L+MH2 differentiated into mature tenocyte-like cells, which expressed tenogenic markers such as *SCXB*, while slightly downregulated the osteogenic marker gene *OCN*, despite the fact that those cells did not possess any of the morphological characteristics of osteoblasts. On the basis of this evidence, we can presume that *SCXB* expression in sheep tendons, especially in fetuses, is able to inhibit osteogenic differentiation regardless of the *OCN* expression. These mechanisms are still to be verified, and the role of OCN-positive cells in sheep tendons must be clarified.

Tendon maturation in sheep not only involved tissue-specific components (tenocytes and ECM), but also the supporting tissues (blood vessels and nerve endings). The amount and organisation of blood vessels, as well as VEGF expression, which is a major angiogenic factor, were both negatively related to aging. Adult tendon has a limited ability to positively modulate the vascular tissue and, because VEGF plays a key role in tendon healing, VEGF gene therapy was applied to improve tendon vascularisation (Ju et al. 2006; Yoshikawa et al. 2006.) Moreover, VEGF165-transfected bone marrow-derived mesenchymal stem cells significantly promoted angiogenesis during graft remodeling of anterior cruciate ligament reconstruction allowing to achieve the best mechanical properties in rabbits (Wei et al. 2011). A close correlation between VEGF and blood vessels in both types of sheep fetal and adult tendons was observed. Intriguingly, only in mid-fetal tendons, VEGF-secreting cells were organised in islets that were positioned close to blood vessels. The strict correlation between VEGF and blood vessels is well documented in several tissue models (Martelli et al. 2009; Barboni et al. 2012b; Mauro et al. 2014). This research confirms that VEGF is a trophic supply for blood vessels. Indeed, a higher VA could be essential to support a highly plastic tissue having a very high synthetic activity especially in fetal tendons. Nevertheless, a role for VEGF in sustaining protection and survival of tendon cells in line with the effects recently reported in human tenocytes (Liang et al. 2012) and chondrocytes (Maes et al. 2012) should not be excluded. Healthy human tenocytes express several VEGF isoforms (Liang et al. 2012), and *VEGFA* mRNA is strongly upregulated following hypoxia under both low- and high-serum conditions. Additionally, VEGF protein released in culture medium increased fourfold by anoxia, exercising a rescue role from cell death (Liang et al. 2012).

There is no detailed study on Achilles/calcaneal tendon innervation, even if the nervous system seems to control different tissue functions. Data demonstrate that afferent (sensory) and efferent endings run into the epitenon, paratenon and endotenon where specific neuronal mediators, which regulate tendon pain, inflammation and tissue homeostasis, are released (Ackermann, 2013). The adult proper tendon itself results practically deprived of neither neuro nor vascular supply (Sharma & Maffulli, 2005). By contrast, in the endotenon of 14 cm sheep fetuses there were large bundles of nerve fibers; these were positioned closely to groups of NGF secreting rounded cells. Tendon NGF expression and the presence of nerve endings progressively decreased, becoming very limited in adult tendon surrounding tissues. The neuronal and vascular dynamism of fetal tendons explains their higher propensity to plasticity (Ackermann, 2013). Mid-gestation stage fetal tendons were characterised by several proteins, such as VEGF, OCN and NGF that were expressed by a group of rounded cells localised in the endotenon, organised in large islets. An organised blood vessel network was localised amongst and below the endotenon rounded cells, arranged in large islets. This type of organisation resembled stem cells niches.

In addition, sheep fetal tissues, unlike adult tendons, contained cells that were able to express pluripotency stem cell markers, such as *TERT*, *SOX2*, *NANOG* and *OCT4A* mRNAs. In addition, through the IHC analysis, it was possible to confirm TERT, SOX2 and NANOG protein expression exclusively in the endotenon. Moreover, in fetal tendons, TERT and SOX2 were excluded from the nucleus and became exclusively cytoplasmic in some cells. These findings confirm this dimorphic subcellular localisation already described for SOX2 (Avilion et al. 2003) and TERT (Russo et al. 2006, 2007). This cytoplasmic translocation may represent early signs of stemness weakening (Mattioli et al. 2012). The presence of these stem-like cells was observed within the endotenon and in proximity to blood vessels, thus suggesting that they might promptly respond to local and systemic regulatory signals, as suggested by Lui (2013) for adult tendon stem cells (TSCs) in their niche. Even if sheep adult tissues did not express pluripotency of stem cell markers, in other animal models the existence of TSCs has been fully demonstrated (Salingcarnboriboon et al. 2003; Bi et al. 2007; Lui, 2013; Mienaltowski et al. 2013). This cell population, usually sorted on CD44 positivity (Ruzzini et al. 2013), exhibits stem cell characteristics and mesenchymal stem cell markers expression, as STRO1 and CD146 together with tenogenic markers, such as α-SMA and TNMD (Bi et al. 2007; Zhang et al. 2010a; Zhang & Wang, 2010b), which make them a distinct stem cell population within the tendon. However, it has been recently demonstrated that age-related variations in human TSCs affect the number of isolated cells and their self-renewal potential (Ruzzini et al. 2013). This finding explains the lower ability of adult tendons to spontaneously and efficiently repair themselves.

In conclusion, during fetal tendon development ovine tendons undergo a series of morphological and biochemical changes that involve both cells and ECM. Indeed, cell nuclei morphology, cellularity, PI, and Cxs 43 and 32 decreased throughout tendon maturation. Moreover, the endotenon was more developed in fetal tendons, especially in the mid-stage. At this stage this tissue was characterised by two classes of cells: large rounded cells organised in groups; and a layer of smaller and more compacted ones. Moreover, in sheep tendons biochemical changes induced a dramatic reduction of ECM molecules, growth factors, such as TGF-β1, VEGF and NGF, as well as blood vessels and nerve fibers in adult tissues. However, except for COL1, all these analyzed components were expressed within the endotenon. In this type of tissue, especially in mid-fetal tendons, there were structures that resembled stem cells niches for their morphology and expression profile. In both types of fetuses, some of the endotenon cells expressed several pluripotency stem cells markers.

For the first time, sheep tendon maturation has been analytically described, clarifying the processes that lead to tendon differentiation. The results provide new data to give a better understanding of the reason for a progressive reduction of the regenerative properties in adult tendons. In addition, the present research supplies additional morphological and biochemical information on ovine tendon that, due to its high translational value, is frequently used as an experimental model to develop new cell-based therapeutic strategies in order to improve tendon healing.
